# Cancer-educated mesenchymal stem cells promote the survival of cancer cells at primary and distant metastatic sites via the expansion of bone marrow-derived-PMN-MDSCs

**DOI:** 10.1038/s41419-019-2149-1

**Published:** 2019-12-09

**Authors:** Buqing Sai, Yafei Dai, Songqing Fan, Fan Wang, Lujuan Wang, Zheng Li, Jingqun Tang, Li Wang, Xina Zhang, Leliang Zheng, Fei Chen, Guiyuan Li, Juanjuan Xiang

**Affiliations:** 1 NHC Key Laboratory of Carcinogenesis and the Key Laboratory of Carcinogenesis and Cancer Invasion of the Chinese Ministry of Education, Xiangya Hospital, Central South University, Changsha, Hunan China; 2 Cancer Research Institute, School of Basic Medical Science, Central South University, Changsha, Hunan China; 3 Hunan Cancer Hospital, The Affiliated Cancer Hospital of Xiangya School of Medicine, Central South University, Changsha, Hunan China; 4 Hunan Key Laboratory of Nonresolving Inflammation and Cancer, Changsha, Hunan 410013 China; 5 Department of Pathology, The Second Xiangya Hospital, Central South University, Changsha, Hunan 410013 China; 6 Department of Thoracic Surgery, The Second Xiangya Hospital, Central South University, Changsha, Hunan 410013 China; 7 Department of Spinal Surgery, The Second Xiangya Hospital, Central South University, Changsha, Hunan 410013 China

**Keywords:** Chemotaxis, Non-small-cell lung cancer, Experimental models of disease

## Abstract

Bone marrow mesenchymal stem cells (BMSCs) are multipotent stromal cells that can differentiate into a variety of cell types. BMSCs are chemotactically guided towards the cancer cells and contribute to the formation of a cancer microenvironment. The homing of BMSCs was affected by various factors. Disseminated tumour cells (DTCs) in distant organs, especially in the bone marrow, are the source of cancer metastasis and cancer relapse. DTC survival is also determined by the microenvironment. Here we aim to elucidate how cancer-educated BMSCs promote the survival of cancer cells at primary tumour sites and distant sites. We highlight the dynamic change by identifying different gene expression signatures in intratumoral BMSCs and in BMSCs that move back in the bone marrow. Intratumoral BMSCs acquire high mobility and displayed immunosuppressive effects. Intratumoral BMSCs that ultimately home to the bone marrow exhibit a strong immunosuppressive function. Cancer-educated BMSCs promote the survival of lung cancer cells via expansion of MDSCs in bone marrow, primary tumour sites and metastatic sites. These Ly6G^+^ MDSCs suppress proliferation of T cells. CXCL5, nitric oxide and GM-CSF produced by cancer-educated BMSCs contribute to the formation of malignant microenvironments. Treatment with CXCL5 antibody, the iNOS inhibitor 1400w and GM-CSF antibody reduced MDSC expansion in the bone marrow, primary tumour sites and metastatic sites, and promoted the efficiency of PD-L1 antibody. Our study reveals that cancer-educated BMSCs are the component of the niche for primary lung cancer cells and DTCs, and that they can be the target for immunotherapy.

## Introduction

Bone marrow mesenchymal stem cells (BMSCs) are multipotent stromal cells that can differentiate into a variety of cell types, including osteocytes, chondrocytes, adipocytes, epithelial cells and endothelial cells^1–3^. BMSCs have been extensively explored in functions of immunomodulation, tissue regeneration and hematopoietic support^4–6^. BMSCs with robust chemotactic properties are recruited to tumours and contribute to cancer progression^7,8^. Fifty-five to sixty-five per cent of injected BMSCs in mice were recovered from the bone marrow, indicating the specific bone marrow homing of systemic BMSC^9^. However, the homing ability of BMSCs was affected by various factors^9,10^. BMSCs are not only the key modulator of bone marrow environment, but also the main component of cancer microenvironment.

The behaviour of cancer cells is strongly influenced by their microenvironment^11^. Meanwhile, tumour cells persistently shape their microenvironment, thereby establishing an abnormal ecosystem. Tumour-associated fibroblasts promote tumorigenesis, whereas normal fibroblasts do not show potent tumorigenesis. These properties indicate that the tumorigenesis of inflammatory fibroblasts is due to the influence of cancer cells^12,13^.Tumour–stroma interactions modulate the microenvironment to be more permissive to cancer cells^14^. Tumour cell hierarchy, as well as multiple cellular elements in the microenvironment, co-evolve during the process of carcinogenesis^11^. When cancer cells leave their primary sites, enter the bloodstream and lodge into a distant organ, they display heterogeneity because of evolutionary selection^15^. BMSCs also evolve and differentiate in cancer microenvironments^16,17^. Once the BMSCs home to a cancer microenvironment in response to chemokines and cytokines secreted by cancer cells, they are ‘educated’ by the cancer microenvironment. BMSCs can differentiate into adipocytes, endothelial and fibroblasts in the cancer microenvironment^1,18^.

Increasing evidence suggests that cancer cell dissemination is an early event, sometimes taking place even before the formation of overt primary tumours^19^. The fate of disseminated tumour cells (DTCs) influences cancer patients outcomes^15^. The survival of DTCs in distant organs is determined by the formation of a pre-metastatic niche^20^. DTCs in distant organs have enhanced malignant features and can redisseminate^19^. DTCs may recirculate from the bone marrow to disseminate again, which is the source of cancer relapse. Bone marrow is a preferred site for DTCs and is considered to be a ‘metastatic niche’^21^. The bone marrow microenvironment is the niche not only for hematopoietic stem cells but also for disseminated cancer cells^22^.

Although the potential for undergoing differentiation of BMSCs into multiple cell type has been validated in vitro, the lack of a MSC-specific marker limits the investigation into the behaviour of BMSCs in cancer patients. In this study, we set out to establish a model that allows us to investigate the dynamic change of BMSCs and how the interaction of BMSCs and cancer cells affect cancer progression. Our study highlights the co-evolution of cancer and stromal compartments. We describe the evolution of BMSCs during the homing process and the progression of cancer by identifying two subpopulations that are distinct from the original BMSCs. We identify the subpopulations of intratumoral BMSCs (T-BMSCs), which show high motility and immunosuppressive effects, and BMSCs, which move from primary tumour sites back into the bone marrow (B-BMSCs) and are associated with an stronger immunosuppressive environment. The cancer-educated BMSCs promote the survival of cancer cells in primary and distant metastatic sites via the induction of bone marrow-derived polymorphonuclear myeloid-derived suppressor cells (PMN-MDSCs) expansion. A better understanding of the features of microenvironmental BMSCs raises the possibility that targeting specific BMSCs can help to prevent the progression of cancer in different stages.

## Methods

### Cells

Murine BMSCs from C57BL/6 mice were purchased from Cyagen Company, China. Non-small cell lung cancer (NSCLC) cell lines including H157, A549, H460 and murine Lewis lung carcinoma cells (LLCs) were cultured in RPMI-1640 medium supplemented with penicillin G (100 U/mL), streptomycin (100 mg/mL) and 10% fetal calf serum. Cells were grown at 37 °C in a humidified atmosphere of 5% CO_2_ and were routinely sub-cultured using 0.25% (w/v) trypsin-EDTA solution. Human BMSCs were obtained from BM aspirates of non-haematological malignant tumour patients. Samples were from Xiangya Hospital, Central South University, China. The collection of the bone marrow was performed for diagnosis. The patients were informed about the sample collection and have signed informed consent forms. Collections and use of tissue samples were approved by the ethical review committees of Xiangya Hospital. Bone marrow aspirates were collected and stored in evacuated tubes containing anticoagulants. Mononuclear cells were isolated from bone marrow using Ficoll-Paque^TM^ PLUS (Density 1.077 ± 0.001 g/mL, GE Healthcare, 17144002). The mononuclear cells were collected and cultured in flasks. Three days after plating, the non-adherent cells were removed. When the adherent cells reached confluence, the cells were then passaged and used for the following experiments at passages 4–6. Human BMSCs and mice BMSCs are monolayer cultured in Medium For Human Mesenchymal Stem Cells (HUXMA-03011-440,cyagen). Firefly luciferase-expressing Luci-LLCs were a kind gift from Professor Wen Zhou (Cancer Research Institute, Central South University). GFP-BMSCs and RFP-LLCs were constructed by lentivirus-mediated green fluorescent protein (GFP) or red fluorescent protein (RFP) gene transduction. The lentivirus-based vector expressing GFP or RFP was purchased from Cyagen, China.

### Animal experiments

Six-week-old male C57BL/6 mice were used to examine allograft tumour growth. Animal experiments were conducted following protocols approved by Central South University, China. 1 × 10^6^ of murine LLC cells with or without murine BMSCs were injected subcutaneously into syngeneic C57BL/6 mice. BMSCs were injected with the same amount of LLCs. The tumours were measured daily using calipers, and their volumes were calculated using the following standard formula: length × width^2^ × 0.5. To measure the disseminated cancer cells in the circulation and in the bone marrow, LLCs were stably transfected with red fluorescent protein *RFP* and BMSCs were stably transfected with *GFP*. The *GFP*-positive and *RFP*-positive cells were recorded after analysis on flow cytometer (BD Biosciences).

To investigate the metastatic cancer cells in vivo, the LLCs were stably transfected with the firefly luciferase gene (*luci-LLCs*) were subcutaneously injected with or without BMSCs into C57BL/6 mice. Non-invasive bioluminescence imaging was performed using Bruker In-Vivo Xtreme II instruments. The mice were intraperitoneally injected with 100 μl of d-luciferin (100 mM). The bioluminescence images were acquired using a charge-coupled device camera 10 min after injection. The primary tumours were cut to avoid signal saturation.

To investigate the chemotactic effect of CXCL5, 6-week-old male C57BL/6 mice were used. Murine *RFP*-positive LLC cells (*RFP*-LLCs; 1 × 10^6^) with murine *GFP*-positive BMSCs (*GFP*-BMSCs) were injected subcutaneously into syngeneic C57BL/6 mice. BMSCs were injected with the same amount of LLCs. Fifteen days after the inoculation of cancer cells, the tumour-bearing mice were intraperitonially injected with CXCL5 neutralizing antibody (1 mg/kg/day, R&D), CXCR2 antagonist (1 mg/kg/day, selleck) or IgG control (1 mg/kg/day, R&D) every 3 days. The tumours were measured daily using calipers and their volumes were calculated using the following standard formula: length × width^2^ × 0.5. The *GFP*-positive and *RFP*-positive cells were recorded after analysis on flow cytometer (BD Biosciences).

For the PD-L1 blockage efficacy experiment, 6-week-old male C57BL/6 mice were used. Murine *RFP*-positive LLC cells (RFP-LLCs; 1 × 10^6^) with murine *GFP*-positive BMSCs (GFP-BMSCs) were injected subcutaneously into syngeneic C57BL/6 mice. BMSCs were injected with the same amount of LLCs. Fifteen days after the inoculation of cancer cells, the tumour-bearing mice were intraperitonially injected with PD-L1 antibody (200 µg/mouse, Bioxcell), 1400 W (2 mg/kg/day, Beyotime, China), CXCL5 neutralizing antibody (1 mg/kg/day, R&D) or granulocyte–macrophage colony-stimulating factor (GM-CSF) antibody (R&D) every 3 days. The tumours were measured daily using calipers and their volumes were calculated using the following standard formula: length × width^2^ × 0.5. The *GFP*-positive and *RFP*-positive cells were recorded after analysis on flow cytometer (BD Biosciences).

### Flow cytometry

Lungs, bone marrow and cancer samples were minced, digested and homogenized. Cells were passed through a 70 µm nylon filter. GFP^+^ and RFP^+^ cells were recorded after analysis on flow cytometer (BD Biosciences). The primary antibodies conjugated with fluorochome were used to detect, sort and purify the target antigens.

Extracellular markers included mouse Percp-cy5.5-CD11b, APC-Ly6G, PE-Ly6C, APC-CD4, PE-cy7-CD8, PE-Gr-1, Hunan PE-CXCR2 and mouse Alexa Fluor 647-CXCR2 were used. All antibodies were purchased from BD Biosciences.

### RNA sequencing

RNA-sequencing (RNA-seq) analysis was performed using BGISEQ-500 platform. Total RNA was isolated and cDNA library was constructed. High-quality clean reads were aligned to the human reference genome using Bowtie2. The expression levels for each of the genes were normalized as fragments per kilobase of exon model per million mapped reads (FPKM) by Expectation Maximization. The differential gene expression analysis was performed by DESeq2. Hierarchical cluster analysis based on log2(ratios) of differentially expressed genes was performed by OmicShare. Interpreting sets of genes was based on GO term enrichment in which genes are classified depending on their functional characteristics.

### Cytokine array analysis

Conditioned media was prepared as described above and applied to a RayBiotech antibody array according to the manual. After incubation with media, the containers were then placed on a shaker and washed three times with 1× Wash Buffer at room temperature. Two millilitres of diluted Streptavidin-horseradish peroxidase was then added to each membrane and incubated at room temperature with gentle shaking for 2 h. Finally, the prepared Chemi Reagent Mix was pipetted onto each membrane. The chemiluminescent signals were captured and the intensity of the dots was quantified using a Bio-Rad ChemiDoc XRS system (Bio-Rad, CA, USA). The resulting images were analyzed using Image J to measure the expression levels of the various targets. A positive control was used to normalize the results from the different membranes being compared.

### Cell mobility

Cell mobility capacity was assessed using Transwell Cell Culture Inserts (8 µm pore size, BD Biosciences, New Jersey, USA) in 24-well plates. A total of 1 × 10^5^ cells in 100 µl of serum-free medium were added to the top chamber. The bottom well contained growth medium with 20% fetal bovine serum. Cells were incubated for 36 h at 37 °C and then the cells that had invaded through the filter pores were fixed with methanol, stained with hematoxylin and observed under a microscope. The number of motive BMSCs were counted from five randomly selected 20× fields for each experiment and averaged.

### Real-time PCR

Total RNA for RNA-seq experiment was used for real-time PCR to confirm the expression of genes. cDNA was synthesized from total RNA using the RevertAid First Strand cDNA Synthesis Kit (Thermo Scientific, Waltham, MA, USA). GAPDH was used as the endogenous control. Quantification PCR was performed according to the indications. Real-time PCR was performed using the Bio-RadIQ^TM5^Multicolor Real-Time PCR detection System (Bio-Rad, Berkeley, CA, USA). Relative mRNA expression levels were calculated by the 2^−ΔΔCt^ method. The primers are listed in Supplementary Table 1.

### Preparation of MDSCs from bone marrow and immunosuppressive assay

The Ly6G^+^ MDCSs were isolated from bone marrow. The anticoagulated bone marrow aspirate was collected from mice that received co-injection of LLC cells and BMSCs. The cell suspension was centrifuged at 12,000 r.p.m. for 5 min. The cell pellet was resuspended in phosphate-buffered saline. The Ly6G^+^ MDSCs were isolated by The BD FACScell sorter. The mouse spleen T cells were isolated with CD90.2 MicroBeads in a magnetic field (Miltenyi Biotec). The ly6G^+^ MDSCs were co-cultured with CD90.2-positive T cells for 3 days. The CD4- and CD8-positive cells were measured by flow cytometry (BD Biosciences).

### Statistical analysis

Data are presented as the mean ± SD from at least three separate experiments. Statistical analyses were performed using GraphPad Prism 5 (GraphPad Software, Inc., CA, USA). Group comparisons were performed using Student’s *t*-test. The survival of tumour-bearing mice was analyzed by Kaplan–Meier. A *p*-value of <0.05 was considered to be significant.

## Results

### BMSCs promote lung cancer cell growth and metastasis

**Fig. 1:**
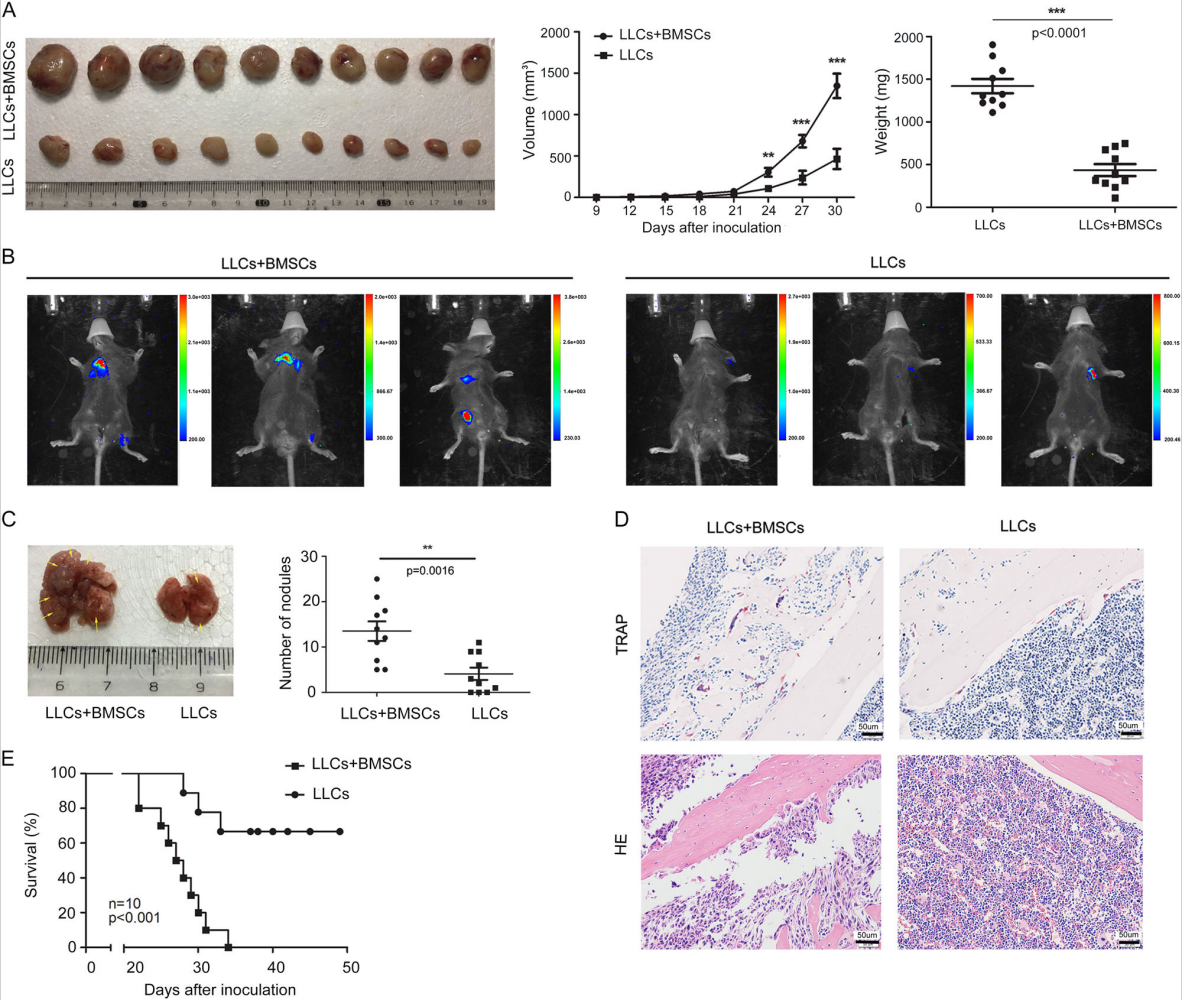
BMSCs promote the growth and metastasis of lung cancer cells. LLC cells, which were stably transfected with firefly luciferase gene (*luci*-LLCs), were subcutaneously injected with or without BMSC into the C57BL/6 mice. Twenty-four days after injection, the allograft tumours were measured. The bioluminescence imaging system was used to monitor the mobility and growth of LLC cells in vivo. **a** Images of allograft tumour growth by LLCs; left, excised tumour; middle, growth curve; right, weight of tumour-bearing mice. Data were analyzed with Student’s *t*-test, **p < 0.01; ***p < 0.001. **b** Bioluminescent images were shown. **c** Representative lung metastatic nodules from mice. The number of metastatic tumour modules formed in the lungs was counted and displayed in scatter plot. Data were analyzed with Student’s *t*-test, **p < 0.01. **d** Representative images of TRAP staining and HE staining of bone section. Amplification: ×100. **e** Kaplan–Meier survival curve for overall survival of mice with co-injection of BMSCs and LLC or injection of LLC alone.

To evaluate whether BMSCs promote cancer growth and facilitate the metastatic process of cancer cells, we constructed syngeneic tumour model that murine BMSCs and murine LLC cells were subcutaneously injected into C57BL/6 mice. LLCs that were stably transfected with the firefly luciferase gene (*luci*-LLCs) were subcutaneously injected with or without BMSCs into C57BL/6 mice. About 24 days later, LLC allograft tumours were significantly larger when the *RFP*-LLCs were co-transplanted with BMSCs (Fig. 1a, *p* < 0.05). The mobility and growth of the LLCs in vivo were examined by bioluminescence imaging. To avoid the signal saturation of the primary tumour, we cut the primary tumour before the detection of in vivo tumour formation. In contrast to the mice that received injection of LLCs alone and that had little evidence of micrometastatic cells, the mice that received injection of a mixture of LLCs and BMSCs showed high luciferase activity in the lungs and bone marrow (Fig. 1b). The number of metastatic tumour modules formed in the lungs was greater in the mixture-injection group than in the LLCs single-injection group (Fig. 1c). Hematoxylin and eosin-stained sections showed metastatic nodules in the lungs in the mixture-injection group (Supplementary Fig. 1A). Histological analysis demonstrated increases in the numbers of Tartrate-resistant acid phosphatase(TRAP)-positive cells and bone lesions (Fig. 1d). Addition of BMSCs shortened the survival of the recipient mice compared to injection of LLCs alone (Fig. 1e). These results indicate that BMSCs promote the growth and metastatic abilities of lung cancer cell.

### Cancer–BMSCs interaction improve capability of dissemination and homing of cancer cells and BMSCs to distant organs

**Fig. 2:**
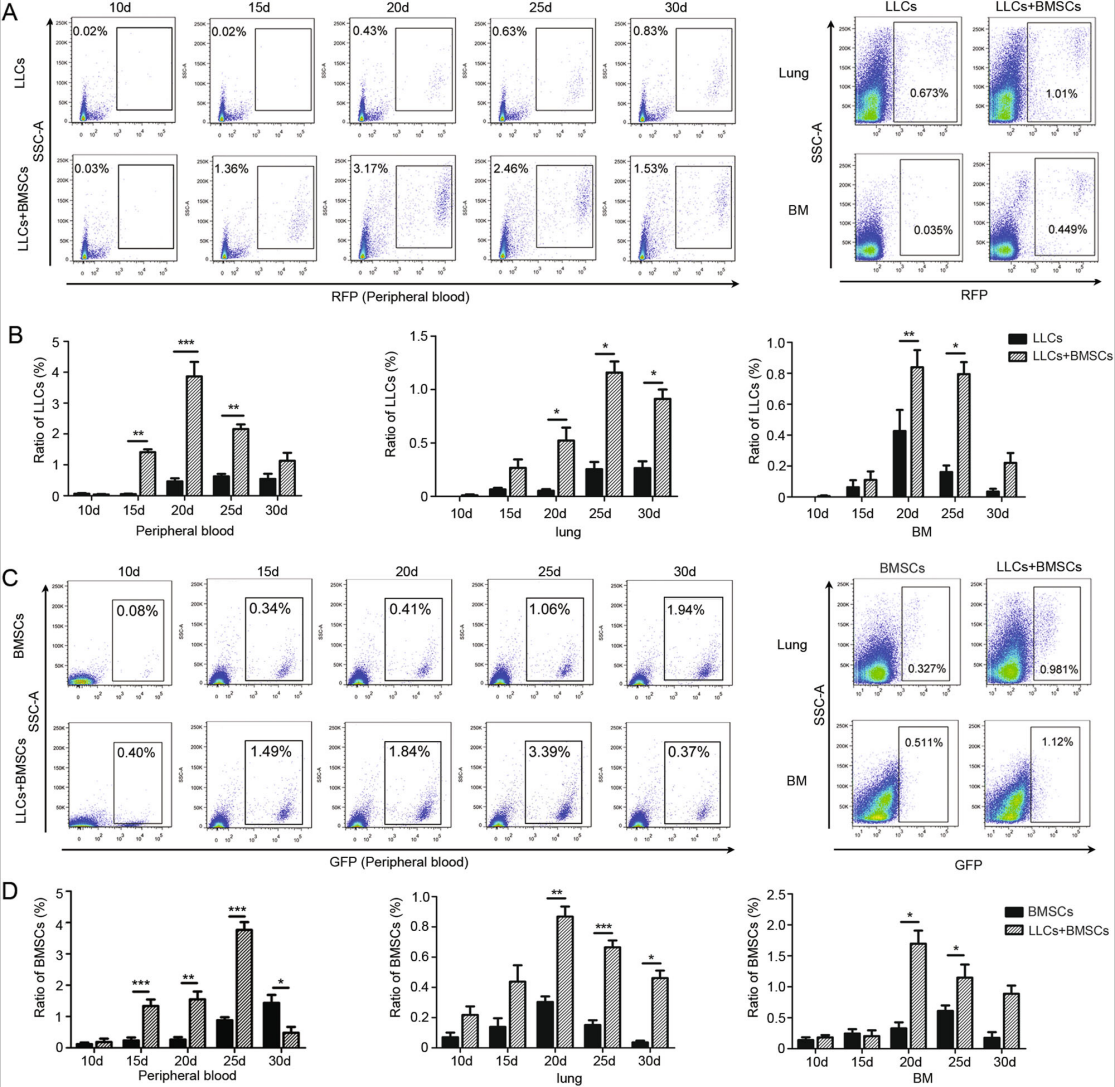
Cancer–BMSCs interaction improve capability of dissemination and homing of cancer cells and BMSCs to bone marrow and lungs. Red fluorescent protein-labelled Lewis lung carcinoma (*RFP*-LLCs) cells were injected subcutaneously with or without green fluorescent protein-labelled BMSCs (*GFP*-BMSCs). The flow cytometry assay was used to measure the *RFP*-positive LLCs and *GFP*-positive BMSCs in circulation, lung and bone marrow in different days after inoculation. **a** The ratio of *RFP*-positive LLCs in peripheral blood, lungs and bone marrow in the mixture-injection group and in the LLC injection alone group. **b** Column bar chart was presented to show the ratio of circulating cancer cells, cancer cells in lungs and bone marrow. **c** The ratio of *GFP*-positive BMSCs in peripheral blood, lungs and bone marrow in the mixture-injection group and in the BMSCs injection alone group. **d** Column bar chart was presented to show the ratio of BMSCs in peripheral blood, lungs and bone marrow. Data were presented as the mean ± SD and analyzed with Student’s *t*-test. *p < 0.05; **p < 0.01; ***p < 0.001.

Dissemination and survival of cancer cells in the bloodstream and in distant organs are required for the formation of metastatic loci. To measure the disseminated cancer cells in the circulation and in bone marrow, *RFP*-labelled LLC cells (*RFP*-LLCs) were injected subcutaneously into C57BL/6 mice with or without *GFP*-labelled BMSCs (*GFP*-BMSCs). The flow cytometry assay revealed that circulating *RFP*-positive LLCs were detected in mice that received the mixture injection or LLCs injection alone, suggesting that the dissemination of cancer cells into the circulation is a frequent event. The ratio of circulating cancer cells in the mixture-injection group was greater than that in the LLC injection alone group (Fig. 2a, b). The modified ISET combined with fluorescent imaging revealed the existence of *RFP*-LLCs in the circulation (Supplementary Fig. 1B). The ratio of *RFP*-positive LLCs residing in the bone marrow and in the lungs was greater in the mixture-injection group compared with the LLC alone injection group (Fig. 2a, b). *GFP*-BMSCs were also detected in the circulation, in bones and in the lungs of the mice that received LLC and BMSC co-injection, indicating that intratumoral BMSCs also disseminated into the circulation (Fig. 2c, d). The number of *RFP*-LLCs in circulation and in bone marrow was positively correlated with the number of *GFP*-BMSCs (Supplementary Fig. 1C). These results indicate that cancer-educated BMSCs preferentially disseminate and home to bone marrow and lungs.

To investigate whether cancer cells affect the dissemination and homing of BMSCs, *GFP*-positive BMSCs were exposed to media collected from LLCs for 72 h, followed by subcutaneous injection into mice. Flow cytometry analysis was used to track the BMSCs in circulation. We found that the BMSCs primed by LLC cells moved earlier into circulation and settled more in the lungs and bone marrow compared with non-primed BMSCs (Supplementary Fig. 1D).

### Spatial evolution of BMSCs during the process of dissemination

**Fig. 3:**
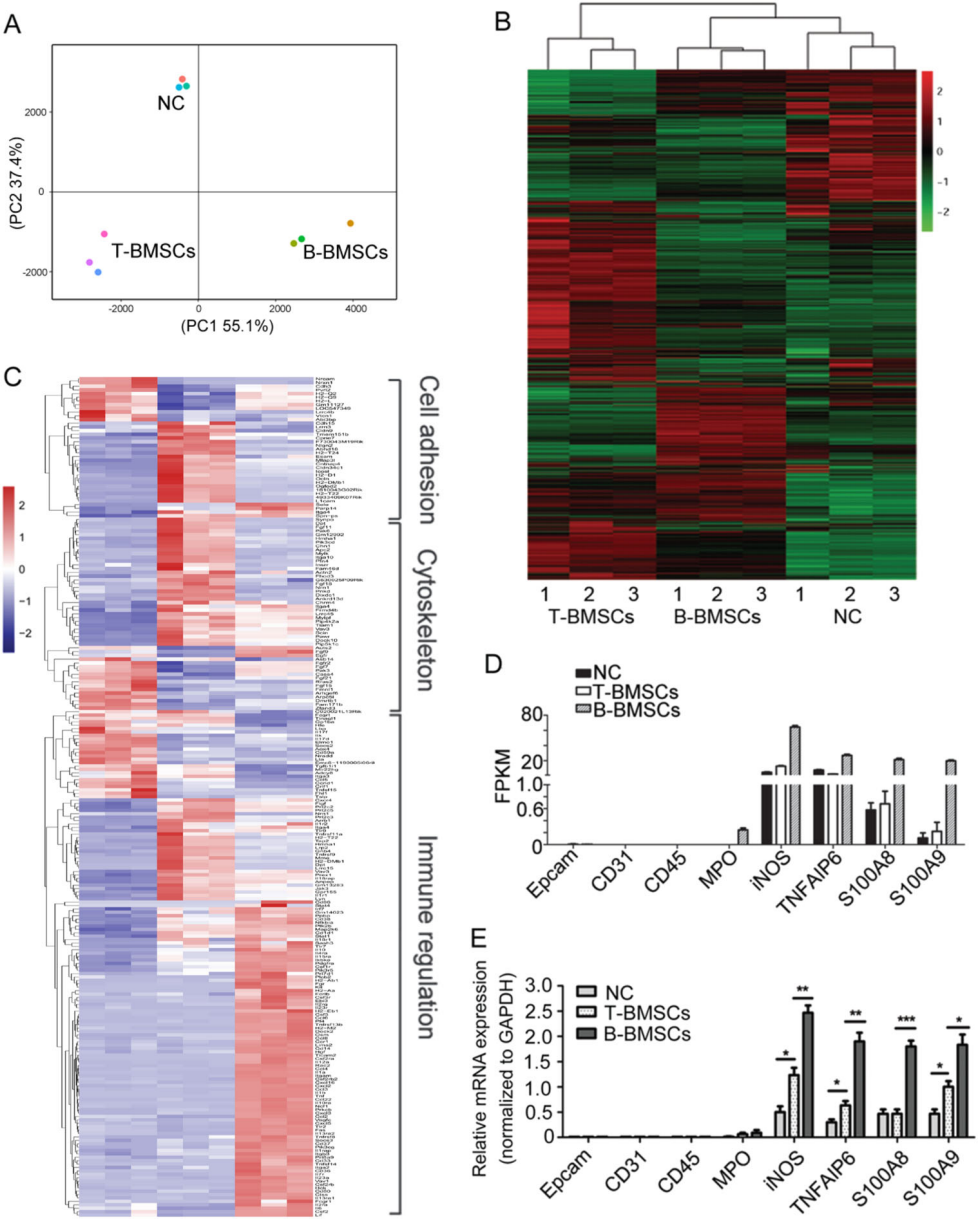
Spatial evolution of BMSCs during the process of dissemination. GFP-BMSCs from the primary inoculation cancer site and bone marrow were collected by flow cytometer sorting technology. The cellular RNA from BMSCs was subjected to RNA-sequencing. **a** Principal component analysis plot. **b** Hierarchical clustering analysis of gene expression. Clustering was performed on differentially expressed genes at FDR < 0.05 from nine samples of BMSCs. Columns represent individual samples and rows represent each gene. Each cell in the matrix represents the expression level of a gene feature in an individual sample. Red and green in cells reflect high and low expression levels, respectively, as indicated in the scale bar (log2-transformed scale). **c** Hierarchical clustering analysis on differentially expressed genes related to selected functions. Columns represent individual samples and rows represent each gene. **d** Expression of genes enriched in the osteoclast differentiation and immunosuppressive pathway quantified by RNA-Seq. FPKM for selected gene transcripts obtained by RNA-Seq. **e** The expression of genes was validated by real-time PCR. Data were analyzed with Student’s *t*-test, *p < 0.05; **p < 0.01; ***p < 0.001.

BMSCs are attracted to cancer sites in response to chemokines produced in the cancer microenvironment. As shown above, the intratumoral BMSCs can disseminate into the circulation and reside in bones. We then compared different transcriptomic signatures by performing RNA-seq. We subcutaneously injected the *RFP*-LLC cells and *GFP*-BMSCs into C57BL/6 mice and then collected GFP-BMSCs from the primary inoculation cancer site and from the bone marrow by flow cytometric sorting technology. The sequencing raw data are available by accession number (GSE120456). Gene expression profiling was performed on BMSCs collected from 3 samples for each group. Principal component analysis showed that the BMSCs collected from primary cancer sites (T-BMSCs) and those from the bone marrow (B-BMSCs) had globally different gene expression profiles (Fig. 3a). Hierarchical clustering analysis showed discrete clustering of the BMSC subsets from cancer primary sites (T-BMSCs) and tumour-bearing bone marrow (B-BMSCs) (Fig. 3b). Compared with intact BMSCs, intratumoral BMSCs had 1820 upregulated and 1147 downregulated genes. mRNA profiling demonstrated that the genes with higher expression in T-BMSCs were cytoskeleton-, cytokine- and immunoregulation-related genes (Fig. 3c and Supplementary Table 2). Compared with the intratumoral BMSCs, cancer-educated BMSCs moving to the bone marrow (B-BMSCs) had 1483 upregulated and 1220 downregulated genes. A Gene Ontology (GO) enrichment analysis demonstrated that the genes with higher expression in B-BMSCs were enriched in immune system process-related genes (Fig. 3c). BMSCs collected from cancer primary sites or from bone marrow were epithelial marker (*EPCAM*), endothelial marker (*CD31*), hematopoietic marker (*CD45* and *CD34*) and neutrophil marker (*MPO*) negative. The upregulated genes were enriched in the osteoclast differentiation and immunosuppressive pathway in B-BMSCs. The T-BMSCs and B-BMSCs expressed many immunologic suppression molecules including *iNOS*^23^, *TNFAIP6*^24^ and *S100A8/S100A9*^25^ (Figs. 3d). The expressions were validated by real-time PCR (Fig. 3e).

### Cancer-educated BMSCs attract cancer cells into circulation through CXCL5/CXCR2

**Fig. 4:**
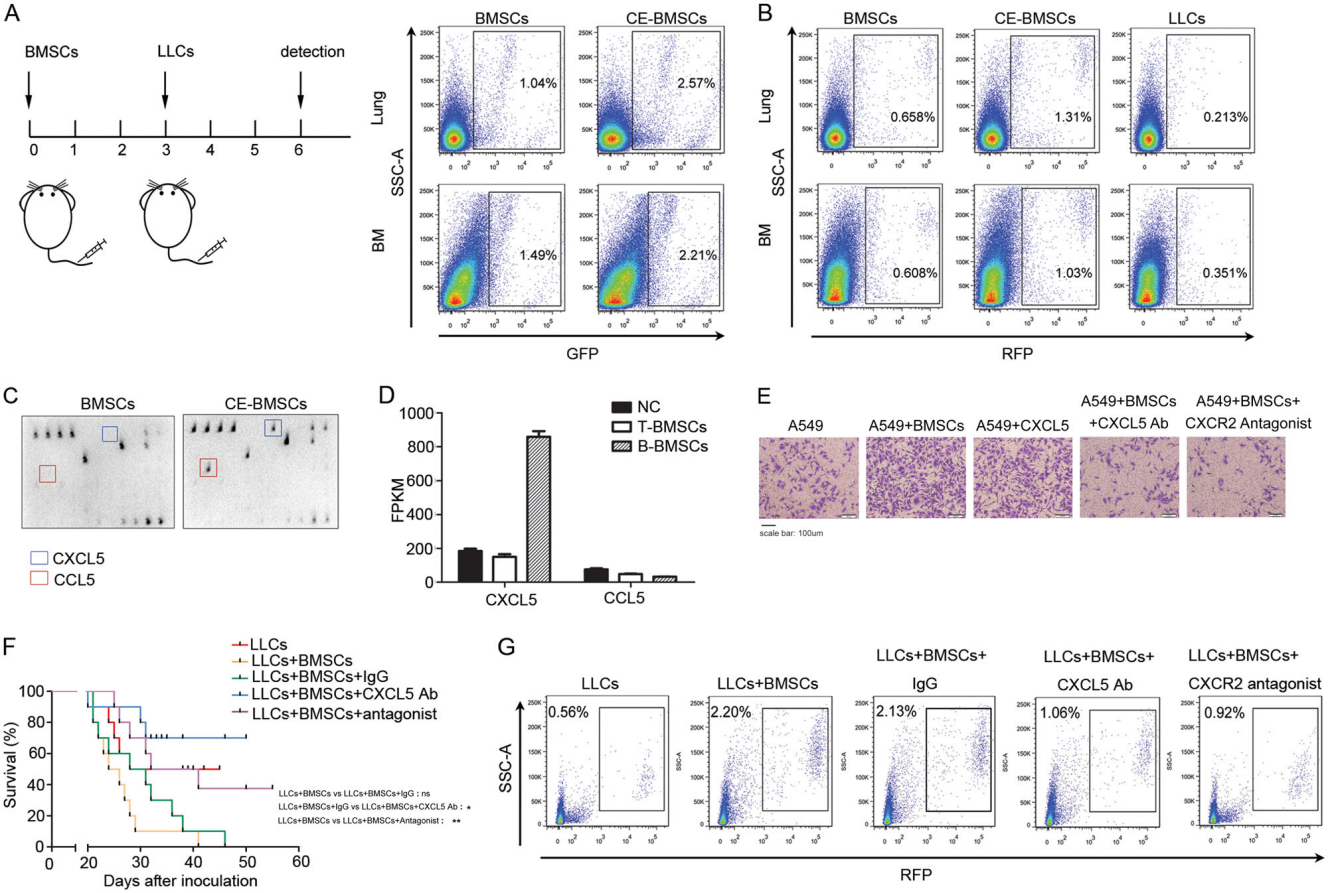
Cancer-educated BMSCs attract cancer cells through CXCL5/CXCR2. *GFP*-BMSCs were co-cultured with LLC for 3 days and then were intravenously injected into C57BL/6 mice. 3 days later, *RFP*-LLC cells were subcutaneously injected. **a** The ratio of *GFP*-positive BMSCs in lung and bone marrow measured by flow cytometry. **b** The ratio of *RFP*-positive LLC cells in lung and bone marrow measured by flow cytometry. **c** Chemokines produced by BMSCs cells. Human BMSCs were co-cultured with A549 lung cancer cells. Three days after co-culture, the media was changed and BMSCs were cultured for additional 3 days. The conditioned media was then collected for Human chemokine proteome profiler antibody array analysis. **d** Expression of *CXCL5* and *CCL5* quantified by RNA-Seq. FPKM for selected gene transcripts obtained by RNA-Seq. Data were presented as the mean ± SD and analyzed with Student’s *t*-test. *p < 0.05; **p < 0.01; ***p < 0.001. **e** Chemotaxis assay was performed by transwell. A549 were co-cultured with human BMSCs and were seeded into transwell insert. Anti-CXCL5 neutralizing antibody or CXCR2 antagonist were added to the underneath reservoir plate. **f** Kaplan–Meier survival curve for overall survival of mice with co-injection of BMSCs and LLC or injection of LLC alone. The mice with co-injection received anti-CXCL5 neutralizing antibody or blockage of its receptor CXCR2 on LLCs before injection. Data were analyzed with Student’s *t*-test, ns: no significance, *p < 0.05; **p < 0.01. **g** The ratio of RFP-positive LLC cells in circulation measured by flow cytometry. RFP-LLCs were injected subcutaneously with or without BMSCs. The flow cytometry assay was used to measure the *RFP*-positive LLCs in circulation. Mice were treated with anti-CXCL5 neutralizing antibody. LLCs were treated with CXCR2 antagonist before its inoculation in the mice. CE-BMSCs: cancer-educated BMSCs.

We next determined whether cancer-educated BMSCs may attract cancer cells into the circulation. We co-cultured BMSCs with LLC cells in vitro and intravenously injected the cancer-educated *GFP*-BMSCs into C57BL/6 mice, followed by intravenous injection of *RFP*- LLCs 3 days later. Compared with the control BMSCs, intravenously injected cancer-educated BMSCs resided more in lungs and bone marrow (Fig. 4a and Supplementary Fig. 2A). Intravenous injection of cancer cell-primed BMSCs attracted more LLCs to the lungs and bone (Fig. 4b and Supplementary Fig. 2B). We then measured differential chemokine secretion, which might account for the differences in the numbers of cancer cells mobilized into the circulation by cancer cell-educated BMSCs vs. non-educated BMSCs. We collected conditional media from human BMSCs alone or BMSCs co-cultured with A549 lung cancer cells. A number of chemokines were significantly upregulated in cancer cell-educated BMSCs compared with non-educated BMSCs, including CXCL5 and CCL5 (Fig. 4c). Having analyzed the gene expression patterns determined by RNA-seq in T-BMSCs and B-BMSCs, we found that T-BMSCs and B-BMSCs exhibited high expression of the genes coding for the chemokines *CXCL5*, but not *CCL5* (Fig. 4d). The expressions of *CXCL5* and *CCL5* were validated by real-time PCR (Fig. 6a). The lung cancer A549 cells, H157 cells, H460 cells and LLCs were shown to be CXCL5 receptor CXCR2 positive (Supplementary Fig. 2C). Recombinant CXCL5 showed a strong chemotactic effect on A549 cells, H157 cells, H460 cells and LLCs (Fig. 4e and Supplementary Fig. 2D, E, F). The chemotactic effects were reversed by anti-CXCL5 neutralizing antibody or CXCR2 antagonist (Fig. 4e and Supplementary Fig. 2D, E, F). The chemotactic role of CXCL5 derived from cancer-educated BMSCs on LLCs was investigated in C57BL/6 mice. C57BL/6 mice were subcutaneously injected with *RFP*-LLCs and BMSCs. Fifteen days later, intraperitoneal injection of anti-CXCL5 neutralizing antibody or CXCR2 antagonist decreased the recruitment of subcutaneously injected *RFP*-LLC cells into the circulation and significantly prolonged the survival of tumour-bearing mice (Fig. 4f, g and Supplementary Fig. 3A).Treatment with anti-CXCL5 neutrlizing antibody and CXCR2 blockage also reduced the growth of primary tumours, indicating that CXCL5/CXCR2 could have effect on tumour growth other than chemotactic effects (Supplementary Fig. 3B, C, D).

### Cancer-educated BMSCs induce the expansion of PMN-MDSCs in bone marrow

**Fig. 5:**
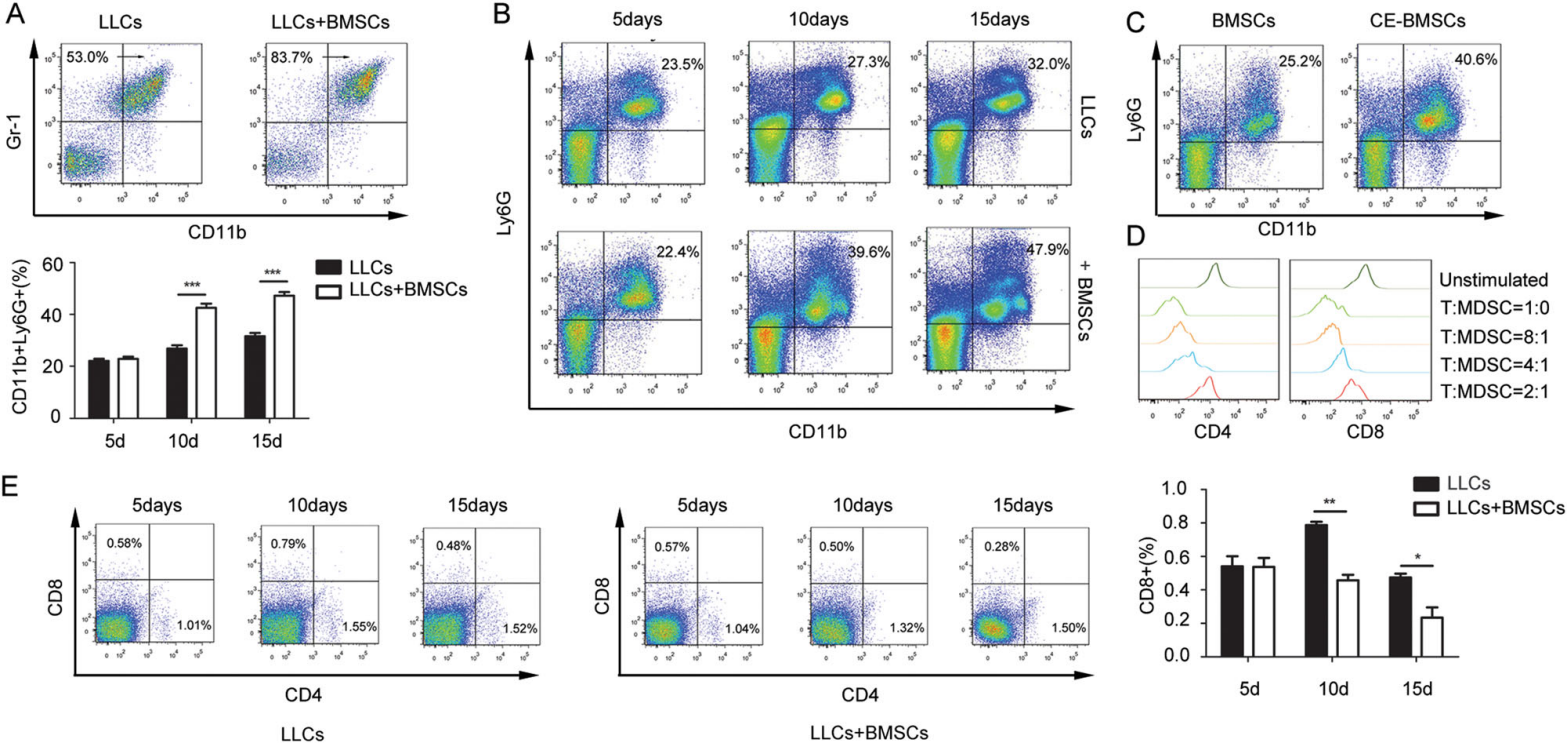
B-BMSCs induce the expansion of PMN-MDSCs in the bone marrow. LLCs and BMSCs were subcutaneously injected in C57BL/6 mice. **a** The ratio of Gr-1^+^/CD11b^+^ cells in the bone marrow of tumour-bearing mice measured by flow cytometry. **b** The ratio of CD11b^+^Ly6G^+^in the bone marrow of tumour-bearing mice measured by flow cytometry. Column bar chart was presented to show the ratio of CD11b^+^Ly6G^+^in the bone marrow. **c** The ratio of CD11b^+^Ly6G^+^ cells in the bone marrow measured by flow cytometry. Tumour-free mice were given intracavitary injections of BMSCs. **d** Bone marrow Ly6G^+^granulocytic cells inhibited CD4^+^ and CD8^+^ T-cell proliferation in vitro. Ly6G^+^ MDSC subpopulations were isolated by magnetic activated cell sorting. T cells were isolated from bone marrow by magnetic activated cell sorting from tumour-free mice. Ly6G^+^ MDSCs were co-culture with T cells isolated from bone marrow in different ratio. After were stimulated by anti-CD3^+^ antibody and anti-CD28^+^ antibody, cells were subjected to flow cytometry to measure the ratio of CD4^+^ and CD8^+^ T cells. **e** CD4^+^ and CD8^+^ T cells in bone marrow were measured by flow cytometry. Bone marrow from mice with co-injection of LLC cells and BMSCs and LLC cells alone were collected. Column bar chart was presented to show the ratio of CD8^+^ T cells in the bone marrow. Data were presented as the mean ± SD and analyzed with Student’s *t*-test. *p < 0.05; **p < 0.01; ***p < 0.001.

To explore the mechanism of how cancer-educated BMSCs promote the survival of lung cancer cells in the primary tumour sites and in bone marrow, we studied immunosuppressive cells in bone marrow in LLC tumour-bearing mice with or without BMSCs. A prominent expansion of Gr-1^+^/CD11b^+^ cells in the bone marrow was found in both groups of tumour-bearing mice, especially in mice with co-injection of LLC cells and BMSCs (Fig. 5a). We further found that among the Gr-1^+^/CD11b^+^ cells, the proportion of CD11b^+^Ly6G^+^Ly6C^lo^ granulocytic cells, but not CD11b^+^Ly6C^+^ monocytic cells, was significantly increased (Fig. 5b and Supplementary Fig. 4A). The proportion of CD11b^+^Ly6G^+^Ly6C^lo^ granulocytic cells in spleen was also significantly increased (Supplementary Fig. 4B). Intracavitary injection of cancer-educated BMSCs into the bone marrow resulted in the expansion of CD11b^+^Ly6G^+^Ly6C^lo^ cells (Fig. 5c). Ly6G^+^ MDSC subpopulations were isolated from bone marrow by magnetic activated cell sorting. These CD11b^+^Ly6G^+^Ly6C^lo^ granulocytic cells potently inhibited CD4^+^ and CD8^+^ T-cell proliferation in vitro (Fig. 5d). CD4^+^ and CD8^+^ T cells in the bone marrow and spleen were obviously reduced in mice with co-injection of LLC cells and BMSCs in contrast to the mice with LLC injection alone (Fig. 5e and Supplementary Fig. 4C). These populations of CD11b^+^Ly6G^+^Ly6C^lo^ cells fit the criteria of PMN-MDSC.

### PMN-MDSC depletion enhances efficacy of anti-PD-L1 treatment

**Fig. 6:**
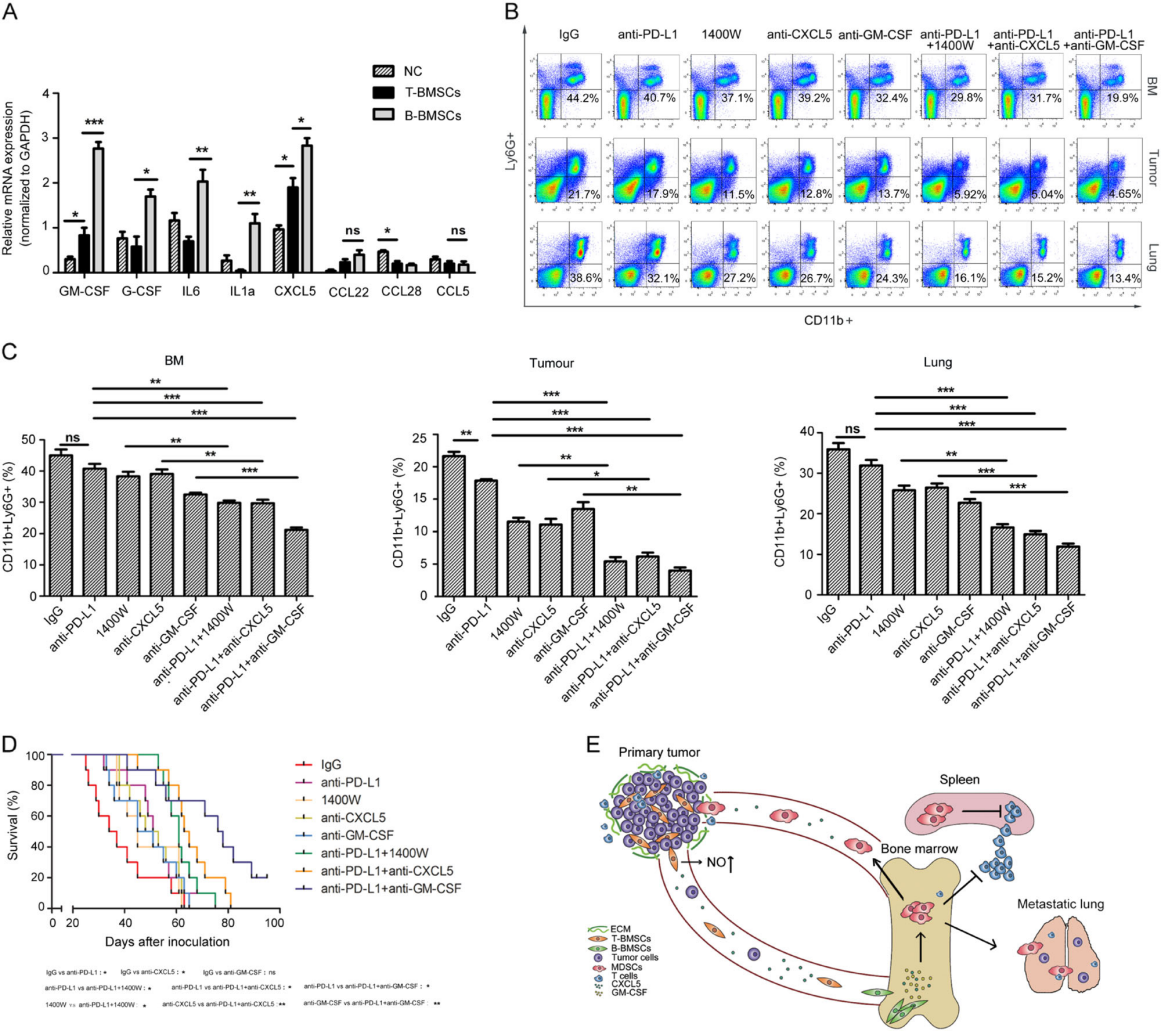
PMN-MDSC depletion enhances efficacy of anti-PD-L1 treatment. **a** Expression of genes that have been implicated in MDSC expansion quantified by real-time PCR. *p < 0.05; **p < 0.01; ***p < 0.001. **b** The ratio of CD11b^+^Ly6G^+^ cells in the bone marrow, lungs and primary tumour sites determined by flow cytometry was shown. Mice were subcutaneously coinjected by LLCs and BMSCs. Tumour-bearing mice were treated with CXCL5 antibody, GM-CSF antibody or iNOS antagonist 1400 W, respectively, or combined with anti-PD-L1 antibody. **c** The ratio of CD11b ^+^ Ly6G ^+^ cells in the bone marrow, lungs or primary tumour sites was presented in column bar chart. Data were presented as the mean ± SD and analyzed with Student’s *t*-test. *p < 0.05; **p < 0.01; ***p < 0.001. **d** Kaplan–Meier survival curve for overall survival of mice with co-injection of BMSCs and LLC. Tumour-bearing mice were treated with PD-L1 antibody combined with CXCL5 antibody, 1400 W or GM-CSF antibody, respectively. **e** Proposed working model of BMSCs in different site of tumour-bearing mice.

We then performed real-time PCR to analyse the expressions of genes that have been implicated in MDSC expansion, including *CXCL5*, *G-CSF*, *IL-6*, *IL-1*, *GM-CSF*, and *iNOS* in T-BMSCs and B-BMSCs (Fig. 6a). We found that *iNOS*, *GM-CSF* and chemokine *CXCL5* were upregulated in T-BMSCs and B-BMSCs (Figs. 6a and 3e). We speculate that cancer-educated BMSCs remodelled the cancer microenvironment through these MDSC-related molecules. C57BL/6 mice were subcutaneously injected with *RFP*-LLCs and BMSCs. Fifteen days after inoculation, intraperitoneal injection of CXCL5 antibody, GM-CSF antibody or iNOS antagonist 1400 W dramatically reduced the accumulation of PMN-MDSCs in the bone marrow, lungs and primary tumour sites compared with IgG-negative control (Fig. 6b). It demonstrated that cancer-educated BMSCs remodel the microenvironment in bone marrow, primary tumour sites and lungs through MDSC-related molecules.

Although a lot of evidences that PD-1/PD-L1 blockage has been shown to be helpful in treatment of advanced lung cancer patients, immunosuppression and immune evasion decreased its clinical efficacy^26–28^. We then sought to investigate if PMN-MDSC depletion enhances efficacy of PD-L1 blockage. C57BL/6 mice were subcutaneously injected with *RFP*-LLCs and BMSCs. Fifteen days after inoculation, the tumour-bearing mice were intraperitonoally injected with anti-PD-L1 mAb. Anti-PD-L1 mAb reduced the primary tumour growth and PMN-MDSCs in primary tumour sites (Fig. 6b, c and Supplementary Fig. 5A-C). In combination with the anti-CXCL5 mAb, 1400 W or anti-GM-CSF mAb, anti-PD-L1mAb reduced PMN-MDSC accumulation in the primary tumours, bone marrow and the lungs more significantly than anti-PD-L1 mAb treatment alone or anti-CXCL5 mAb, 1400 W or anti-GM-CSF mAb treatment alone (Fig. 6b, c). The combination of CXCL5 antibody, 1400 W or GM-CSF antibody with anti-PD-L1mAb resulted in increased number of T cells in primary tumour sites (Supplementary Fig. 5D, F). The combination of CXCL5 antibody, 1400 W or GM-CSF antibody with anti-PD-L1 mAb reduced primary tumour growth and *RFP*-positive LLCs in lungs and prolonged the survival of cancer bearing mice compared with PD-L1 antibody alone, indicating that MDSC depletion can enhance the efficacy of immunotherapy (Fig. 6d and Supplementary Fig. 5A, B, E, F).

## Discussion

The present work aimed at providing a better understanding of the roles of stromal cells in cancer cell growth and metastasis. We found a spatial evolution of BMSCs during the process of dissemination. We identified two types of BMSCs, each exhibiting different characteristics in mobility and immunologic regulation. T-BMSCs, which reside in the primary cancer, are highly mobile and immunosuppressive. B-BMSCs, which move from the primary cancer to the bone marrow, acquire the adverse characteristic of immunologic inhibition. The immunosuppressive molecules produced by cancer-educated BMSCs induce expansion of PMN-MDSCs and affect the efficacy of PD-L1inhibitory therapy (Fig. 6e).

During cancer progression, novel genotypic and phenotypic variants emerge via gene mutation or changes in gene expression^29^. Tumour cells and their stroma co-evolve^30^. The stroma evolves as a direct response to stress. In this study, we clarified the spatial evolution of BMSCs during cancer progression. The classification of cancer-educated BMSCs is based on gene expression patterns. These BMSC variants facilitate the adaptive evolution of cancer cells. The stroma cells affect not only the primary cancer cells but also metastatic cancer cells by remodelling the distant organs.

In the bone marrow niche, hematopoietic stem cells and DTCs usually remain in the quiescent G0 state^31^. Although the dissemination of cancer cells occurred much earlier than we expected, DTCs in the bone marrow are not immediately competent to initiate growth^32^. Circulating Tumor Cells (CTCs) in the circulation have a short half-life, persisting for only 1–2 h. Most CTCs die in the circulation as a result of shear stress and/or anoikis^33,34^.

Growth factors and neovasculature in the bone marrow affect the reactivation of dormant cancer cells. The dormant DTCs may be reactivated by the osteoclast-mediated release of bone-derived growth factors^35^. When blood vessels begin to sprout, the new tips produce molecules that transform dormant cancer cells into metastatic tumours^36^. This process transforms a dormant niche into a metastatic niche. A subpopulation of BMSCs with both endothelial and pericytic cell surface markers suppresses the homing of cancer cells to the bone marrow^31^. Although it has been long known that BMSCs are an important component of the hematopoietic stem cell niche, no specific markers have been identified. BMSCs are heterogeneous and their surface markers and behaviour are constantly changing. In contrast to the BMSCs that reside in the bone marrow, the BMSCs that reside in the primary cancer sites recruited from the bone marrow showed marked and distinct changes in their gene profiles and other features. In contrast to the original BMSCs, cancer-educated BMSCs that move back into the bone marrow showed characteristics of osteoclasts and immune suppressive cells. These subtypes of BMSCs secreted specific cytokines and chemokines, increasing the recruitment of cancer cells into circulation and bone marrow and increasing their half-life.

MDSCs are generated in the bone marrow from common myeloid progenitor cells. The accumulation and expansion of MDSCs in the bone marrow of tumour-bearing hosts was reported in many studies^37–39^. There are two major populations of MDSCs: PMN-MDSCs and M-MDSCs. In most types of cancer, PMN-MDSCs represent 70–80% of the total MDSC population^40^. Expansion of PMN-MDSCs in the bone marrow, primary tumour sites and metastatic sites mediated by cancer-educated MSCs was observed in our study. GM-CSF^41^, granulocyte-CSF^42^, and macrophage-CSF^43^ are responsible for the development of MDSCs. The pathologic activation of MDSCs in this study was induced by persistent stimulation coming from cancer-educated MSCs. Myeloid cells generated under these conditions are unable to differentiate effectively into mature myeloid cells^40^. PMN-MDSCs and neutrophils share a similar phenotype and morphology^40^. PMN-MDSCs induced by cancer-educated MSCs suppress T-cell function, a mechanism by which cancer cells protect themselves from elimination by the immune system. MDSCs also play important roles in limiting the effect of cancer immunotherapeutics^40^. The roles of MDSCs in limiting the anti-tumour immune response and the effectiveness of immune checkpoint inhibitors are increasingly apparent. Suppression of MDSCs by GM-CSF antibodies or 1400 W enhanced the anti-cancerous effect of PD-L1 antibodies.

## Supplementary information

Supplementary Table 1

Supplementary Table 2

Supplementary Figure 1

Supplementary Figure 2

Supplementary Figure 3

Supplementary Figure 4

Supplementary Figure 5
