# Supplementary material for: Cancer-educated mesenchymal stem cells promote the survival of cancer cells at primary and distant metastatic sites via the expansion of bone marrow-derived-PMN-MDSCs
Source: Cell Death Dis. 2019 Dec 9;10(12):941. doi: 10.1038/s41419-019-2149-1 (PMC6901580; doi:10.1038/s41419-019-2149-1)
Supplement: Supplementary file 1 — Supplementary Table 1 [file 41419_2019_2149_MOESM1_ESM.docx]

| Gene | Forward primer (5’- to 3’) | Reverse primer (5’- to 3’) |
| --- | --- | --- |
| Epcam | GCGGCTCAGAGAGACTGTG | CCAAGCATTTAGACGCCAGTTT |
| CD31 | ACGCTGGTGCTCTATGCAAG | TCAGTTGCTGCCCATTCATCA |
| CD45 | ATGGTCCTCTGAATAAAGCCCA | TCAGCACTATTGGTAGGCTCC |
| MPO | AGTTGTGCTGAGCTGTATGGA | CGGCTGCTTGAAGTAAAACAGG |
| iNOS | GTTCTCAGCCCAACAATACAAGA | GTGGACGGGTCGATGTCAC |
| TNFAIP6 | GGGATTCAAGAACGGGATCTTT | TCAAATTCACATACGGCCTTGG |
| S100A8 | AAATCACCATGCCCTCTACAAG | CCCACTTTTATCACCATCGCAA |
| S100A9 | ATACTCTAGGAAGGAAGGACACC | TCCATGATGTCATTTATGAGGGC |
| CXCL5 | TCCAGCTCGCCATTCATGC | TTGCGGCTATGACTGAGGAAG |
| CCL5 | GCTGCTTTGCCTACCTCTCC | TCGAGTGACAAACACGACTGC |
| GM-CSF | GGCCTTGGAAGCATGTAGAGG | GGAGAACTCGTTAGAGACGACTT |
| G-CSF | ATGGCTCAACTTTCTGCCCAG | CTGACAGTGACCAGGGGAAC |
| IL6 | TAGTCCTTCCTACCCCAATTTCC | TTGGTCCTTAGCCACTCCTTC |
| IL1a | CGAAGACTACAGTTCTGCCATT | GACGTTTCAGAGGTTCTCAGAG |
| CCL22 | AGGTCCCTATGGTGCCAATGT | CGGCAGGATTTTGAGGTCCA |
| CCL28 | GTGTGTGGCTTTTCAAACCTCA | TGCATGAACTCACTCTTTCCAG |
| GAPDH | AGGTCGGTGTGAACGGATTTG | TGTAGACCATGTAGTTGAGGTCA |

Supplementary Table 1 The primers for PCR reactions
