# Supplementary material for: Cancer-educated mesenchymal stem cells promote the survival of cancer cells at primary and distant metastatic sites via the expansion of bone marrow-derived-PMN-MDSCs
Source: Cell Death Dis. 2019 Dec 9;10(12):941. doi: 10.1038/s41419-019-2149-1 (PMC6901580; doi:10.1038/s41419-019-2149-1)
Supplement: Supplementary file 2 — Supplementary Table 2 [file 41419_2019_2149_MOESM2_ESM.docx]

Supplementary Table 2: Functional Clustering of subtypes of BMSCs

| Functional clustering | Number of genes | Gene names |
| --- | --- | --- |
| cell adhesion | 38 | Nrcam,Nrxn1,Cdh3,Pvrl2,H2-Q2,H2-Q9,H2-L,Gm11127,LOC547349,Lrrc4b,Vtcn1,Abi3bp,Cdh15,Lrrn3,Cldn9,Tmem151b,Cpne7,F730043M19Rik，Nlgn2, Abhd18, H2-T24, Esam, Mfap3l,Cntnap4,Cldn34c1,Icosl,H2-D1,Ocln,H2-DMb1,Ogfod2,1810043G02Rik,H2-T22,4933409K07Rik,L1cam,Sele,Parp14,Itga4,Spn-ps |
| cytoskeleton | 51 | Synpo,Dpt,Fgf11,Pak6,Gm12992,Hmha1,Pik3cd,Chn1,Apc2,Mylk,Itga10,Pfn4,Insrr,Fam46d,Actn2,Fhod3,G630025P09Rik,Fgf18,Nrn1,Pnkd,Dixdc1,Ankrd13d,Chrm4,Itga4,Frmd4b,Lrrc45,Mylpf,Pip4k2a,Tiam1,Vav3,Scin,Pawr,Dock10,Pip5k1c,Auts2,Fgf9,Egfr,Asb14,Fgfr2,Fgf7,Pak3,Cass4,Fgf21,Rras2,Fgf15,Fmnl1,Arhgef6,Arpc5l,Dmrtb1,Fam171b,Zfand3 |
| immune regulation | 149 | C920021L13Rik,Fcgrt,Tinagl1,Gp1ba,Hfe,Lbp,Il17f,Itk,Il17d,Elmo1,Socs2,Aox4,Cd59a,Nradd,Lta,Emc8-1190005i06rik,Tgfb1i1,Mir22hg,Adcy8,Itga3,Ccl5,Ccnd1,Crlf1,Tnfsf15,Fhl1,Tslp,Cxcr4,Figf,Prl2c2,Prl2c5,Nrn1,Prl2c3,Arrb1,Il1r2,Itga4,Tlr9,Tnfrsf11a,H2-T22,Tap2,Hmha1,Lrp2,Gnb4,Tnfrsf9,Mme,H2-DMb1,Dpt,Lrrc15,Vav3,Prex1,Il18rap,Anpep,Gm13283,Jak3,Gpr155,Il1r1,Lyn,Cd86,Stat4,Irf7,Gm14023,Ppbp,Cd38,Nfkbia,Ptk2b,Map2k6,Cd1d1,Stat1,Il18r1,Sash3,Tlr7,Il10,Il4ra,Il15ra,Ikbke,Pdgfra,Csf1r,Pik3r5,Prl7d1,Plcb2,H2-Ab1,Fgr,Kit,H2-Aa,Fcrlb,Csf3r,Ebi3,Il2rg,Il23r,H2-Eb1,Csf3,Ccl6,Pf4,Tnfrsf13b,H2-M2,Dock2,Osm,Ccl8,Ccr1,Lims2,Cd14,Hgf,Ticam2,Csf2ra,Il12a,Rac2,Ccl4,Il1a,Itgam,Csf2rb2,Cxcl16,Cxcl2,Ccl3,Il1b,Tnf,Ccl22,Il10ra,Ncf1,Prkcb,Cxcl3,Ccl2,Vegfc,Cxcl5,Tlr2,Fas,Il13ra2,Tnfrsf8,Socs3,Cd37,Pik3cg,Il1rap,Itgb3,Prl8a9,Cd33,Tnfsf14,Itga2,Cd36,Il7r,Il23a,Vav1,Csf2rb,Hck,Cd80,Ctss,Il13ra1,Fcgr1,Il2ra,Il6,Csf2,Lif |
