# Supplementary figures and images for: Cancer-educated mesenchymal stem cells promote the survival of cancer cells at primary and distant metastatic sites via the expansion of bone marrow-derived-PMN-MDSCs
Source: Cell Death Dis. 2019 Dec 9;10(12):941. doi: 10.1038/s41419-019-2149-1 (PMC6901580; doi:10.1038/s41419-019-2149-1)

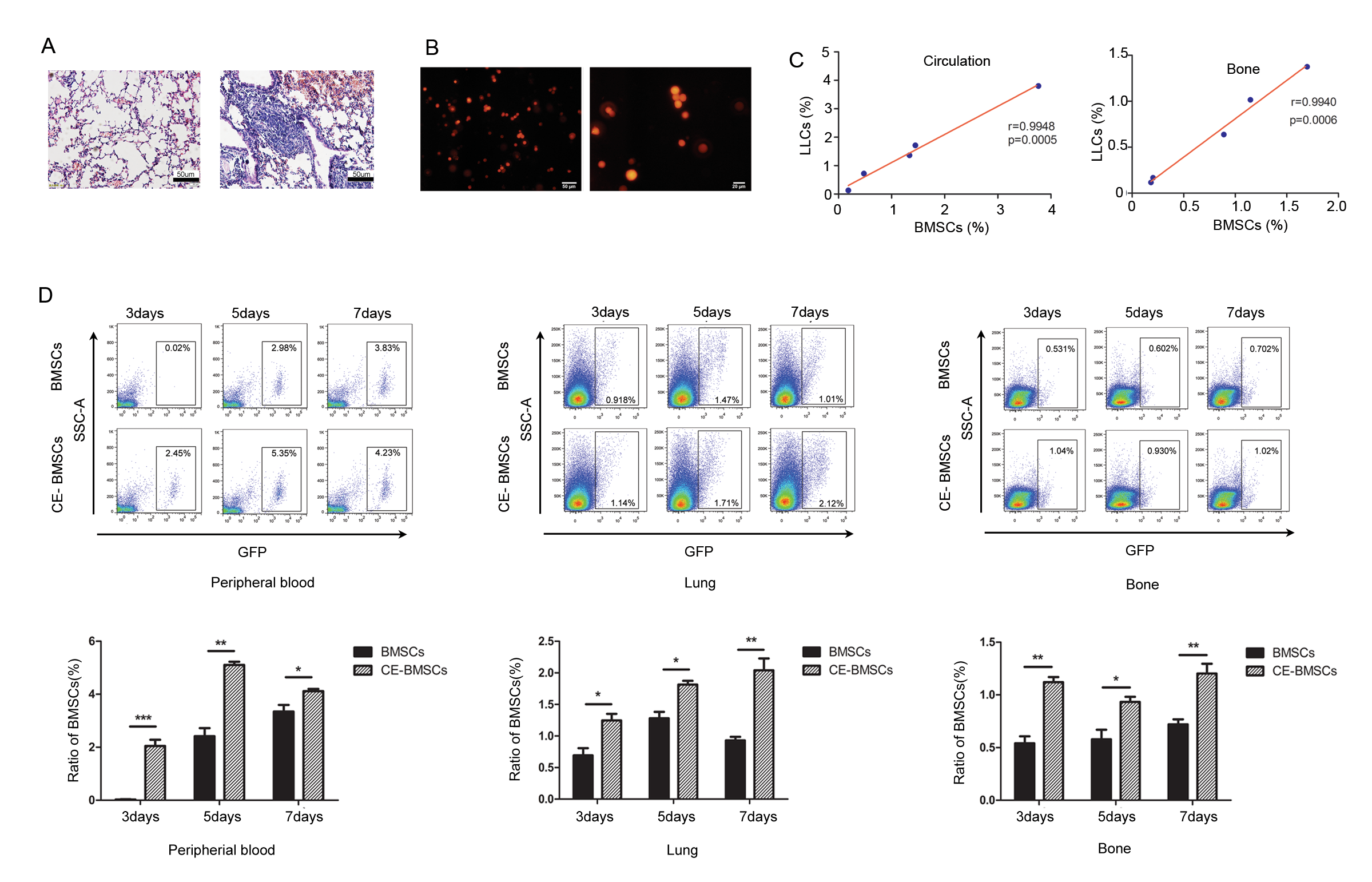

Supplement: Supplementary file 3 — Supplementary Figure 1 [file 41419_2019_2149_MOESM3_ESM.tif]

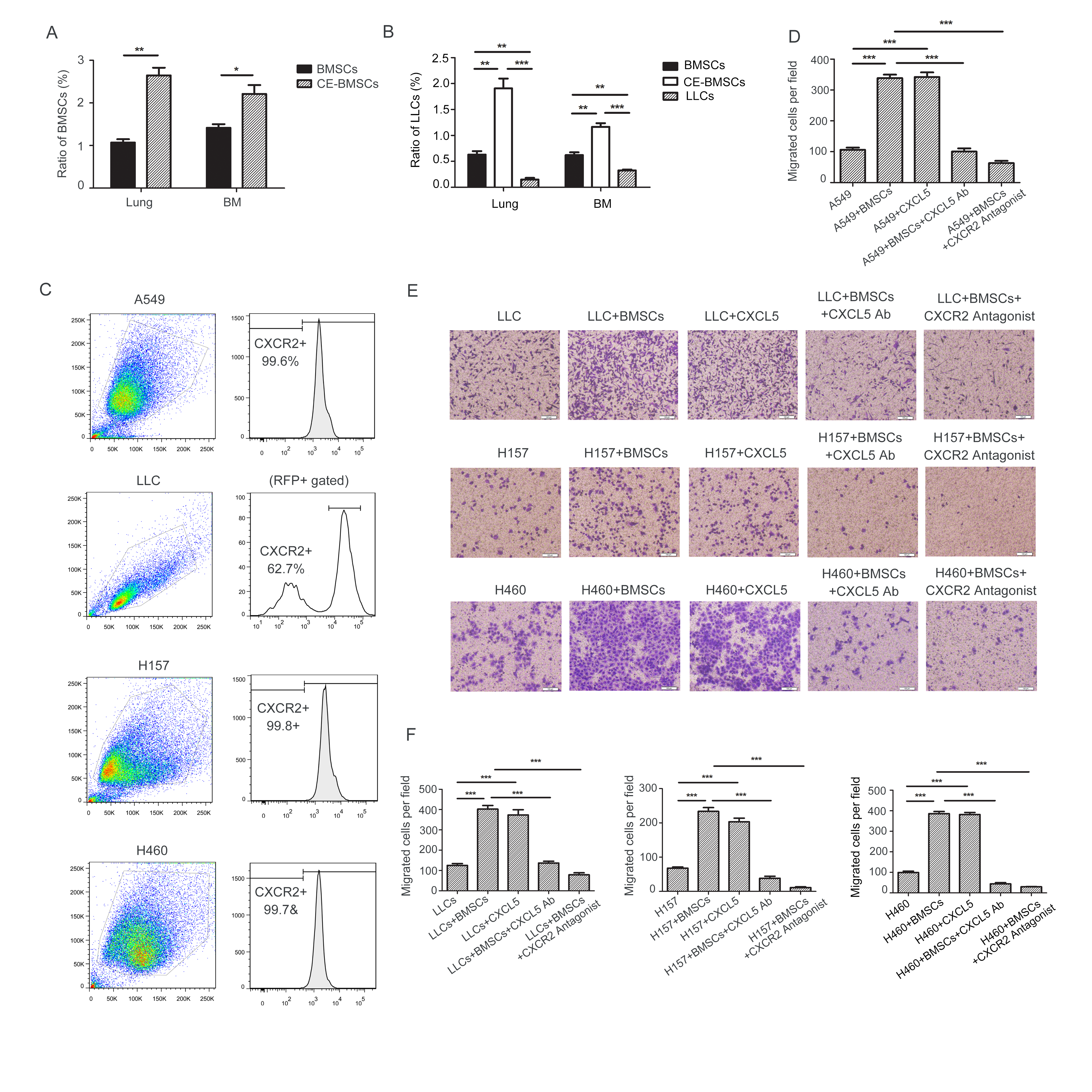

Supplement: Supplementary file 4 — Supplementary Figure 2 [file 41419_2019_2149_MOESM4_ESM.tif]

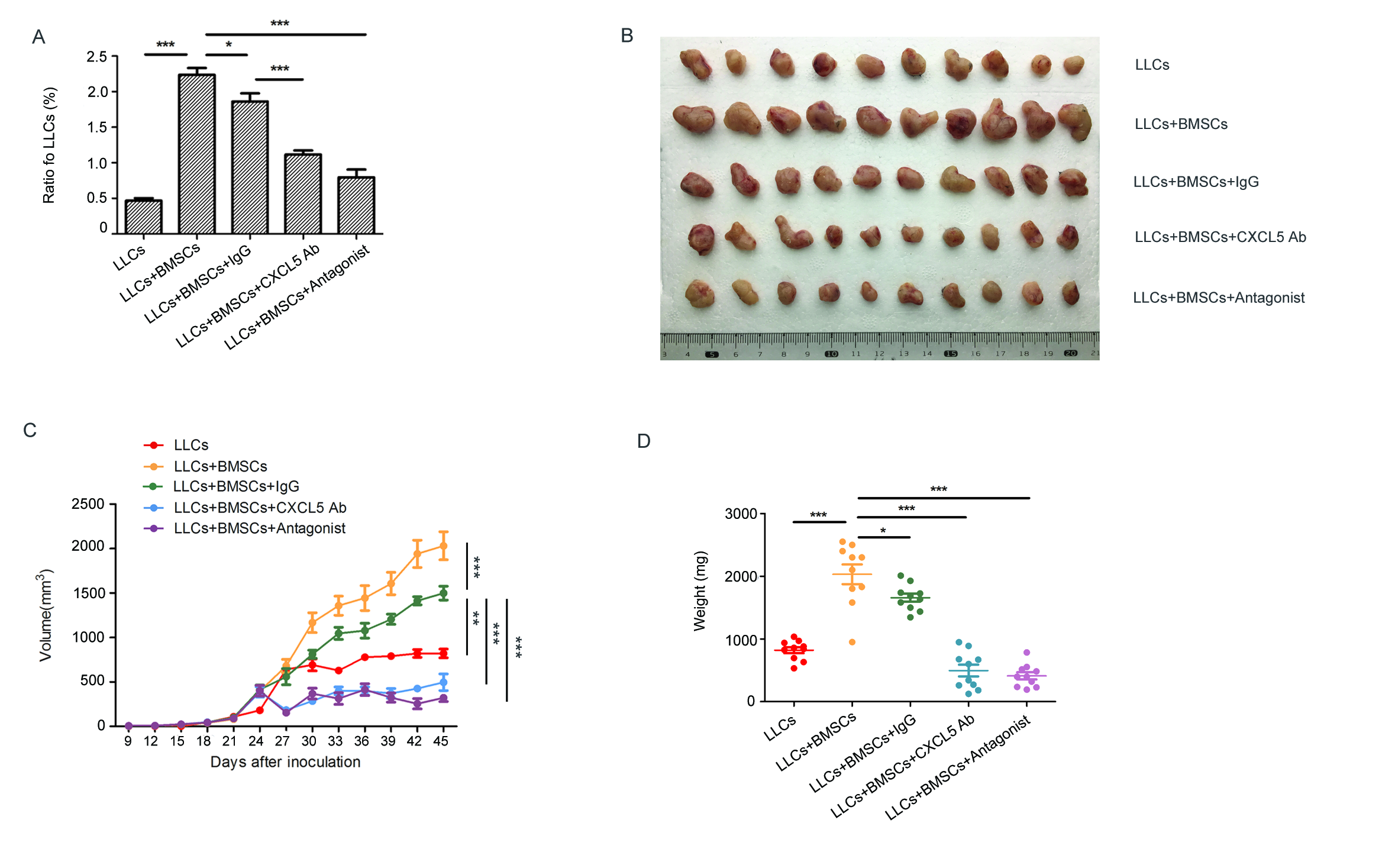

Supplement: Supplementary file 5 — Supplementary Figure 3 [file 41419_2019_2149_MOESM5_ESM.tif]

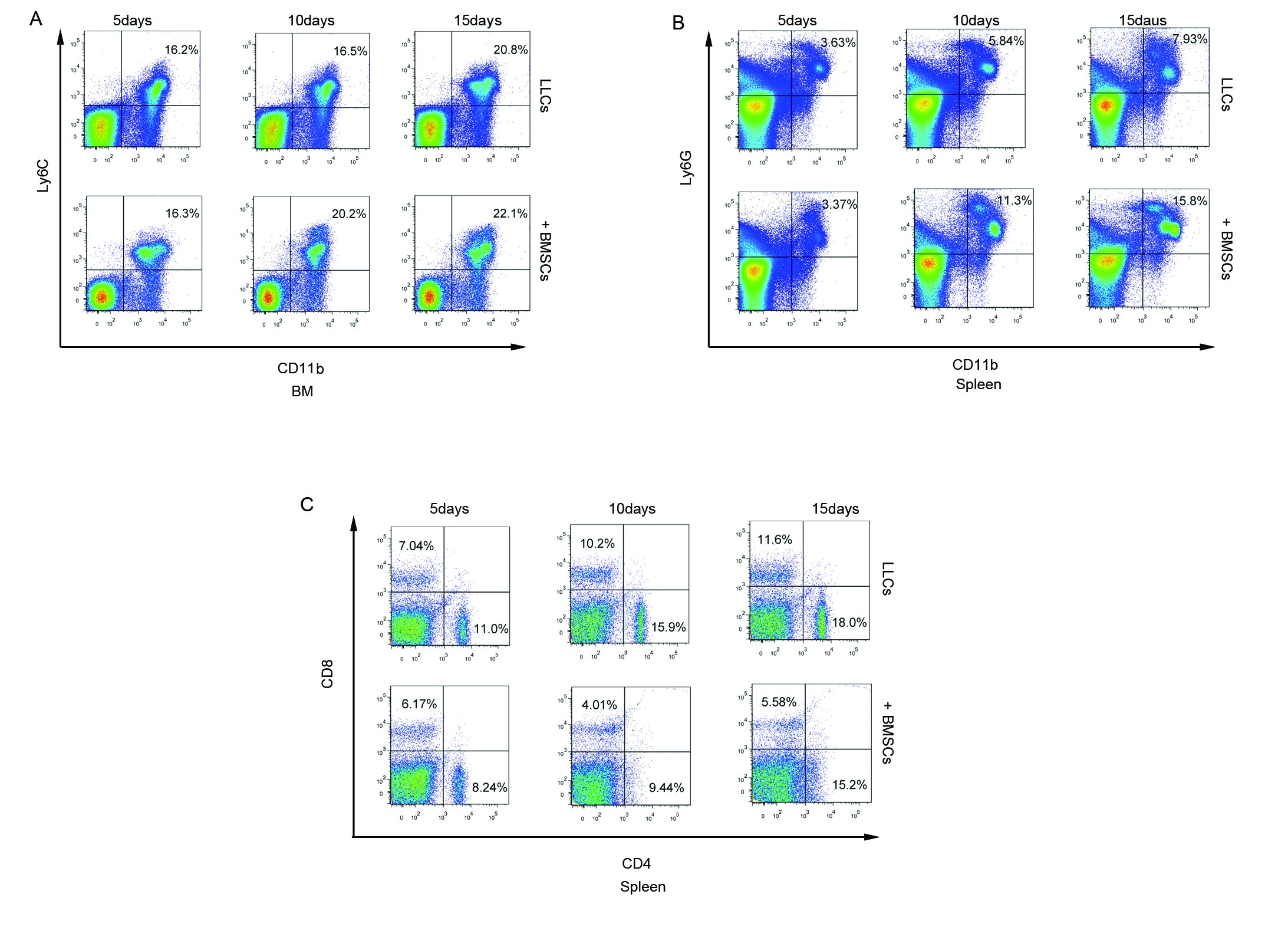

Supplement: Supplementary file 6 — Supplementary Figure 4 [file 41419_2019_2149_MOESM6_ESM.tif]

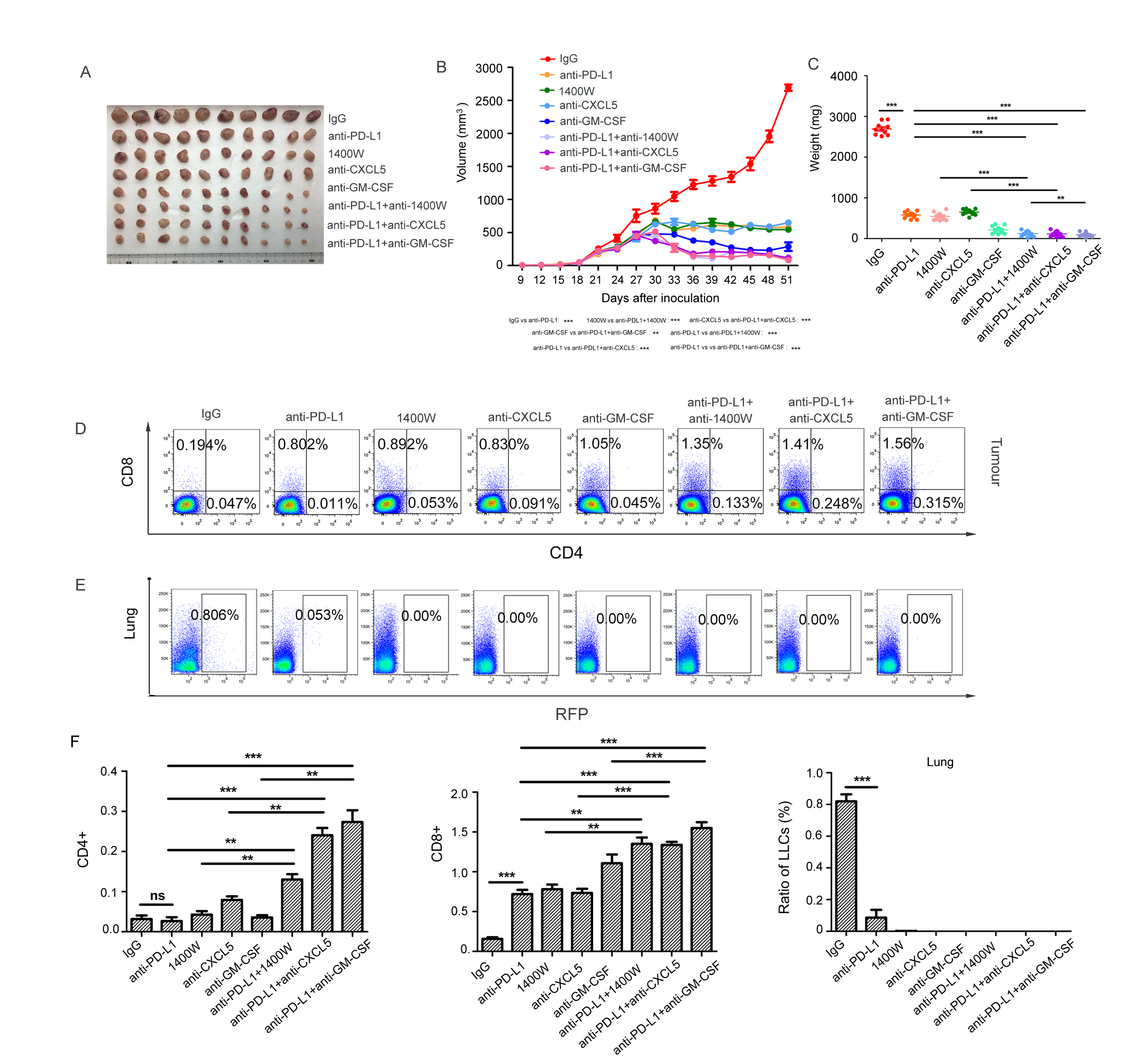

Supplement: Supplementary file 7 — Supplementary Figure 5 [file 41419_2019_2149_MOESM7_ESM.tif]
